# Practical tips for paediatricians: implementing cognitive behavioural therapy for adolescent insomnia

**DOI:** 10.1093/pch/pxag029

**Published:** 2026-04-28

**Authors:** Tasnim Youssef, Rafiaa Valji

**Affiliations:** Department of Chemistry, Faculty of Science, University of Alberta, Edmonton, AB, Canada; Division of Respiratory Medicine, Department of Pediatrics, Faculty of Medicine and Dentistry, University of Alberta, Edmonton, AB, Canada

**Keywords:** Insomnia, DSWPD, Adolescents, CBTi, Sleep hygiene

## Abstract

Insomnia, a sleep condition defined as persistent difficulties initiating or maintaining sleep, affects up to 40% of adolescents, with most continuing to experience symptoms once they begin. It is linked with mood disturbance, academic impairment, fatigue, and reduced quality of life. Despite its significance, few adolescents seek care on their own, and parents frequently underestimate the issue, highlighting the importance of pediatricians in early detection and treatment. Insomnia must be distinguished from delayed sleep–wake phase disorder (DSWPD), a circadian rhythm condition presenting similar complaints but requiring different treatments. Cognitive behavioural therapy for insomnia (CBT-I) is the first-line treatment recommended for adults and increasingly supported in adolescents. CBT-I integrates behavioural and cognitive strategies to target perpetuating factors and has demonstrated meaningful, durable improvements in sleep outcomes. CBT-I can be delivered in groups, individually, or digitally. Pediatricians can direct access to suitable interventions and offer families guidance on core sleep principles.

## Introduction

Insomnia symptoms affect up to 40% of adolescents (1), with nearly 9 in 10 continuing to struggle once symptoms start (2). Insomnia is defined as difficulty falling asleep, staying asleep, or waking too early at least three nights per week for 3 months or longer, with associated functional impairments such as fatigue, poor concentration, academic difficulties, mood disturbance, or somatic complaints (3,4). It frequently co-occurs with other medical, psychiatric, and sleep conditions (5). Its impact on quality of life is profound, linked to reduced self-esteem, depressive symptoms, and poor school performance (6). Few adolescents seek care independently (1), and parents often underestimate the severity of their adolescents' sleep problems (7). Pediatricians, therefore, play a critical role in identifying insomnia early and initiating appropriate management.

## Differential diagnosis

Delayed sleep–wake phase disorder (DSWPD) is characterized by a habitual shift toward later bedtimes and wake times, driven by pubertal changes in circadian regulation and social factors (8). Many adolescents with DSWPD present with difficulty falling asleep and significant morning sleepiness, which can mimic or coexist with insomnia (3). DSWPD is typically managed with circadian-based interventions designed to shift the sleep–wake schedule, such as phase advancement (gradually moving bedtime and wake time earlier) or phase delay/chronotherapy (gradually shifting bedtime and wake time later) (8). Practical clue: if a teen falls asleep and sleeps well when allowed a late schedule—such as on weekends or holidays—suspect DSWPD rather than primary insomnia.

The differential diagnosis also includes restless leg syndrome, which presents with an urge to move the legs or uncomfortable leg sensations at rest, particularly at bedtime as well as periodic limb movement disorder, which is characterized by disruptive leg movements during sleep (9). Obstructive sleep apnea disturbs sleep through repeated arousals and is suggested by habitual snoring, apneas, gasping, choking, or restless sleep. Poor sleep hygiene and insufficient sleep opportunities are also common contributors in adolescence, particularly in the setting of erratic sleep schedules, late caffeine use, evening screen exposure, inadequate time allotted for sleep, or sleep-disruptive bedroom environments (9). Depression, anxiety disorders, and attention-deficit/hyperactivity disorder are also important considerations.

## How to treat insomnia

Cognitive behavioural therapy for insomnia (CBT-I) is recommended by the American Academy of Sleep Medicine and the European Sleep Research Society as the first-line treatment for adults (10,11), and there is now growing evidence for its use in adolescents (4). CBT-I combines education about sleep hygiene with structured behavioural strategies—such as sleep restriction, stimulus control, and relaxation training—alongside cognitive techniques to address unhelpful beliefs about sleep (12). These interventions target the behavioural and cognitive processes that perpetuate insomnia.

Recent systematic reviews and meta-analyses have shown that CBT-I for adolescents with insomnia improves sleep latency, sleep efficiency, subjective sleep quality and insomnia symptoms (13,14). In adults, network meta-analyses confirm CBT-I is more effective than pharmacotherapy for key outcomes, and equally effective when used in combination (15). Despite this, CBT-I remains underutilized, with many clinicians still emphasizing sleep hygiene advice or medications (16). Although melatonin is commonly viewed as a first-line option, its use should be considered adjunctive, given its modest effects in otherwise healthy youth and the high variability in melatonin content across commercial products (17,18).

Limited availability of healthcare providers trained in evidence-based insomnia interventions remains a major barrier to wider CBT-I implementation (19). As a result, families often turn to pediatricians and family physicians for insomnia support, placing this care within an already overburdened primary care setting.

For pediatricians, the key goal is to recognize insomnia early, consider alternative or coexisting diagnoses, counsel families on core behavioural principles, and connect patients to appropriate resources. Table 1 summarizes CBT-I strategies in practical terms (20). Unfortunately, evidence-based digital CBT-I options for adolescents remain limited, with most platforms only occurring in research settings. The DOZE app is a Canadian smartphone-based adolescent sleep health program informed by CBT-I; in a school-based study of 223 high school students, it was associated with more regular sleep schedules, modestly increased total sleep time, and high acceptability (21) (resource: https://www.dozeapp.ca/). Sleepio is a publicly available digital CBT-I program with some relevant youth research, although it is currently intended for adults aged 18 years and older who are being supervised by a clinician (22) (resource: https://www.sleepio.com/sleepio/nhs/391#1/1). In addition to specialized sleep clinics, CBTi practitioners who treat adolescents can be identified through the Canadian Sleep Research Consortium CBT-I Map (resource: https://www.researchsleep.ca/cbtimap).

**Table 1:** Core CBT-I strategies in practical terms for adolescents.

CBT-I strategy	What it is	How it's done
**Sleep Restriction**	Consolidates sleep by limiting time in bed to actual sleep time, then gradually increasing as efficiency improves.	Keep a 1 to 2 week sleep diary to estimate average sleep time. Restrict time in bed to that amount (e.g., 6.5 h if that's the average). Anchor with a fixed wake time. Once sleep efficiency ≥85 to 90%, increase time in bed by 15 to 20 min each week until optimal sleep is reached. Don’t restrict sleep to <6 h/night.
**Stimulus Control**	Strengthens the bed–sleep association and breaks the link with wakefulness.	Go to bed only when sleepy. Use bed only for sleep (no phones, homework, or lying awake). If awake >15 to 20 min, leave the bed, do a quiet/dim-light activity, then return when sleepy. Wake at the same time daily regardless of sleep amount. Avoid daytime naps.
**Sleep Hygiene**	Healthy lifestyle and environmental habits that promote sleep readiness.	Keep consistent sleep and wake times, including weekends. Avoid caffeine/energy drinks late. Limit screen use before bed. Make the bedroom cool, quiet, and dark. Encourage daily exercise (not within 2 h of bedtime).
**Relaxation Training**	Reduces physical and mental arousal that can delay sleep onset.	Practice relaxation strategies before bed: deep breathing, progressive muscle relaxation, guided imagery, or mindfulness. Develop a nightly “wind-down” routine (quiet reading, calm music, stretching). Use relaxation apps or recordings.
**Cognitive Restructuring**	Identifies and replaces unhelpful thoughts about sleep.	Teach adolescents to notice negative thoughts (e.g., “I’ll never sleep”), challenge them (“I usually fall asleep, just not right away”), and replace with more balanced thoughts (“Even if I don’t sleep immediately, my body is resting and I’ll cope tomorrow”). Encourage journaling or thought records.

## Practical strategies

Practical Action Box (Take-Home for Pediatricians)

- **Screen efficiently:** Utilize the validated **Adolescent Insomnia Questionnaire (AIQ)**, a 13-item tool designed for adolescents aged 11 to 18, to identify insomnia symptoms (23).
- **Differentiate:** If sleep is normal when teens are allowed a late schedule (e.g., weekends), suspect delayed sleep–wake phase rather than primary insomnia.
- **Counsel briefly:** Reinforce one or two CBT-I principles (e.g., *leave bed if awake >20 min*, *anchor a consistent wake time*).
- **Refer and resource:** If no improvement despite initial interventions and digital CBTi tools, connect families to CBTi trained psychologists or specialized sleep clinics.
